# Zonisamide Enhances Neurite Elongation of Primary Motor Neurons and Facilitates Peripheral Nerve Regeneration *In Vitro* and in a Mouse Model

**DOI:** 10.1371/journal.pone.0142786

**Published:** 2015-11-16

**Authors:** Hideki Yagi, Bisei Ohkawara, Hiroaki Nakashima, Kenyu Ito, Mikito Tsushima, Hisao Ishii, Kimitoshi Noto, Kyotaro Ohta, Akio Masuda, Shiro Imagama, Naoki Ishiguro, Kinji Ohno

**Affiliations:** 1 Division of Neurogenetics, Center for Neurological Diseases and Cancer, Nagoya University Graduate School of Medicine, Nagoya, Japan; 2 Department of Orthopedic Surgery, Nagoya University Graduate School of Medicine, Nagoya, Japan; 3 Department of Hand Surgery, Nagoya University Graduate School of Medicine, Nagoya, Japan; University of Szeged, HUNGARY

## Abstract

No clinically applicable drug is currently available to enhance neurite elongation after nerve injury. To identify a clinically applicable drug, we screened pre-approved drugs for neurite elongation in the motor neuron-like NSC34 cells. We found that zonisamide, an anti-epileptic and anti-Parkinson’s disease drug, promoted neurite elongation in cultured primary motor neurons and NSC34 cells in a concentration-dependent manner. The neurite-scratch assay revealed that zonisamide enhanced neurite regeneration. Zonisamide was also protective against oxidative stress-induced cell death of primary motor neurons. Zonisamide induced mRNA expression of nerve growth factors (BDNF, NGF, and neurotrophin-4/5), and their receptors (tropomyosin receptor kinase A and B). In a mouse model of sciatic nerve autograft, intragastric administration of zonisamide for 1 week increased the size of axons distal to the transected site 3.9-fold. Zonisamide also improved the sciatic function index, a marker for motor function of hindlimbs after sciatic nerve autograft, from 6 weeks after surgery. At 8 weeks after surgery, zonisamide was protective against denervation-induced muscle degeneration in tibialis anterior, and increased gene expression of *Chrne*, *Colq*, and *Rapsn*, which are specifically expressed at the neuromuscular junction. We propose that zonisamide is a potential therapeutic agent for peripheral nerve injuries as well as for neuropathies due to other etiologies.

## Introduction

Motor paralyses due to damage to the peripheral and central nervous systems, especially those due to motor nerve damage, cause serious disabilities in ADL (activities of daily living), but no effective treatment has been established for this condition. Local administration of neurotrophins, such as nerve growth factor (NGF) and brain-derived neurotrophic factor (BDNF), induces the axonal elongation of motor nerves and ameliorates motor deficits in model animals [1–4]. Similarly, laminins and cadherin-11 promote the axonal elongation of motor neurons [5–8]. These molecules, however, cannot be easily used in clinical settings, because local administration would be the only available option and their half-lives in our body are very short [9–11]. Recently, cell transplantation from different sources has been gaining attention as a therapy for nerve damage and therapeutic effects have been reported in model animals [12–18]. Cell transplantation is thus likely to be one of the promising modalities to treat motor nerve damage, if safety concerns are addressed [19]. Small molecule compounds that were reported to improve motor nerve damage include ibuprofen, a non-steroidal anti-inflammatory drug [20]; valproic acid, an anti-epileptic drug [21]; and Y27632, an inhibitor of Rho-associated kinase (ROK) [22]. Although these are promising compounds, none has yet been available in clinical settings.

Drug repositioning is a strategy for discovering new efficacies of existing drugs whose dosage, administration method, safety margin, adverse effects, etc., are already known, which enables fast application of the drug in clinical practice. Drug repositioning may drastically reduce the cost and time of drug development compared to conventional approaches [23, 24].

In this study, we screened 1,186 FDA-approved drugs to identify a clinically applicable compound that promotes neurite elongation of cultured motor neurons. Firstly, we performed extensive phenotypic screening with NSC34, a neuroblastoma and spinal motor neuron hybrid cell line. We then examined whether candidate compounds identified by the first screening promoted neurite elongation of mouse primary motor neurons. We finally identified that zonisamide, an anti-epileptic and anti-parkinsonian drug, enhanced neurite elongation and increased the number of branch points in primary motor neurons. Zonisamide also improved the viability of H_2_O_2_-treated primary motor neurons and induced mRNA expression of *Bdnf*, *Ngf*, *Ntf4*, *Ntrk1*, and *Ntrk2*. In addition, zonisamide enhanced nerve regeneration in a mouse model of sciatic nerve autograft. We propose that zonisamide is a promising compound for peripheral nerve injuries and neuropathies due to other etiologies.

## Materials and Methods

### Ethics statement

All animal experiments were approved by the Animal Care and Use Committee of Nagoya University. Mice were used to isolate primary spinal motor neurons at embryonic day 13.5 (E13.5) and to examine the effect of intragastric administration of zonisamide after sciatic nerve autograft.

### Culture of NSC34 cells

NSC34 cells (mouse neuroblastoma-spinal cord hybrid cells displaying a multipolar motor neuron-like phenotype) that stably express the doxycycline-induced green fluorescent protein (GFP) construct (NSC34-pTetR12-TO/GFP) [25] were kindly provided by Dr. Shinsuke Ishigaki, Department of Neurology, Nagoya University Graduate School of Medicine. NSC34 cells were cultured in a humidified atmosphere of 95% air-5% CO_2_ in a 37°C incubator in Dulbecco's Modified Eagle's Medium (DMEM, Invitrogen) supplemented with 10% fetal bovine serum (FBS, Thermo Scientific). Cells were seeded at 1.6 × 10^3^ cells/well on a 96-well plate with the differentiation medium of DMEM/F12 (Invitrogen) containing 1% FBS and 2 mg/ml doxycycline to induce GFP expression. On day 1, the differentiation medium was changed to DMEM/F12 (Invitrogen) containing 1% NEAA (Non-Essential Amino Acid, MP Biomedicals) and 2 mg/ml doxycycline, and the cells were incubated for 48 additional hours in the presence of 10 μM of 1,186 FDA-approved chemical compounds (Prestwick Chemical). Then, the nuclei of the cells were stained with the fluorescent dye Hoechst 33342 (1:100,000, Sigma Aldrich), and images of 16 fields per well were automatically taken with the ArrayScan VTI HCS Reader (Thermo Scientific Cellomics). The average neurite lengths in each well were automatically analyzed with the Neuronal Profiling v4.0 BioApplication (Thermo Scientific Cellomics).

### Culture of primary spinal motor neurons

Primary cultures of mouse embryonic spinal motor neurons were dissociated from embryonic spinal cord at E13.5 of C57BL/6J mice. Zonisamide was first dissolved in 100% dimethyl sulfoxide (DMSO) to make 200 μM to 2 mM solutions, and then added to the culture medium at a final concentration of 1 to 20 μM. Culture media with or without zonisamide all contained 0.5% DMSO. Cells were seeded at 4.0 × 10^3^ cells/well on a 96-well plate coated with poly-L-lysine and laminin (Asahi Techno Glass), and maintained in Sumilon neuron culture medium (Sumitomo Bakelite). Cells were incubated for 2 days and were fixed for 15 min with 4% paraformaldehyde in phosphate-buffered saline (PBS) at room temperature. Cells were washed in PBS once, blocked by 2% goat serum and 0.1% Triton-X in PBS, and then immunostained with mouse anti-tau-1 monoclonal antibody (1:500, MAB3420, Millipore) and the Alexa Fluor 555 goat anti-mouse secondary antibody (1:1000, Life Technologies). Neurites were automatically analyzed with the ArrayScan VTI HCS Reader, as stated above. The 200 longest neurites were considered to quantify neurite length and the number of neurite branch points. In addition, the number of cells with neurite length > 25 μm was counted in each well to calculate the ratio of neurite-bearing cells.

### Scratch assay

Primary motor neurons were seeded at 8.0 × 10^4^ cells/cm^2^ × 0.7 cm^2^/well in the Nun Lab-Tek Chamber Slide (Thermo Scientific) coated with poly-L-lysine, and were cultured in Sumilon neuron culture medium for 48 h. Neurites of primary motor neurons were wounded with a linear scratch by a 200-μl sterile pipette tip. The cells were immediately rinsed with PBS and cultured for 48 additional hours in Sumilon neuron culture medium with 0, 1, 10, or 20 μM zonisamide. The cells were then fixed in 4% paraformaldehyde and immunostained with neuron-specific ß-III tubulin monoclonal antibody (1:1000, MAB1195, R&D systems) and with the Alexa Fluor 488 goat anti-mouse secondary antibody (Life Technologies). Images of the scratched areas were taken with a microscope (Olympus LX71), and the length of regenerated neurites of the primary motor neurons at the scratched area was automatically measured by the MetaMorph software (Universal Imaging).

### Time-lapse imaging of axonal elongation of primary motor neurons

Primary motor neurons were seeded at 6.0 × 10^3^ cells in each well of a 96-well plate and cultured in Sumilon neuron culture medium to induce neurite elongation. Phase contrast microscopic images were automatically taken using the IncuCyte ZOOM Live Cell Imaging System (Essen Bioscience) every 8 h for 3 days. Neurite length was automatically analyzed with the IncuCyte’s NeuroTrack software.

### MTS assay

Cell viability was estimated with the MTS assay (CellTiter 96 Aqueous One Solution Cell Proliferation Assay, Promega), which represents mitochondrial reductase activity. Primary motor neurons were initially cultured in Sumilon neuron culture medium for 24 h, and then switched to DMEM/F12 with 0.5% of FBS for 24 h. Variable concentrations (2 to 20 μM) of zonisamide were added to the medium. After 1 h, cells were exposed to 100 μM H_2_O_2_ for 24 h. The cells were incubated with MTS reagent for 2 h and MTS signals were quantified with a microplate reader (PowerScan HT, DS Pharma Biomaterial). MTS signals were normalized to those without H_2_O_2_ treatment.

### Total RNA extraction and real-time RT-PCR analysis

Total RNA was isolated using Trizol (Thermo Fisher Scientific) from primary motor neurons cultured with 0, 1, or 10 μM zonisamide. Total RNA was similarly isolated from a 3-mm segment of the sciatic nerve, and from the tibialis anterior muscle. First strand cDNA was synthesized with ReverTra Ace (Toyobo). We quantified mRNA expression of *Bdnf*, *Ngf*, *Ntf4*, *Ntrk1*, *Ntrk2*, *Map2*, *Mapt*, *Gap43*, *Chrne*, *Colq*, and *Rapsn* using LightCycler 480 Real-Time PCR (Roche) and SYBR Green (Takara). mRNA levels were normalized to *Gapdh*. The primer sequences are shown in S1 Table.

### Western blotting

Zonisamide (0, 10, 20 μM) was added to primary motor neurons 4 h after starting the cultures. The cells were lysed on day 2 or 3 with a buffer containing 50 mM 4-(2-hydroxyethyl)-1-piperazineethanesulfonic acid (HEPES) pH 7.0, 150 mM NaCl, 1% glycerol, 1% Triton X-100, 1.5 mM MgCl_2_, 1 mM ethylene glycol tetraacetic acid (EGTA), 100 mM NaF, 10 mM sodium pyrophosphate, 1 mg/ml aprotinin, 1 mg/ml leupeptin, 1 mg/ml pepstatin A, 1 mM phenylmethylsulfonyl fluoride (PMSF), and 1 mM sodium orthovanadate. Total proteins were dissolved in 1x Laemmli buffer, separated on a 10% sodium dodecyl sulfate (SDS) polyacrylamide gel, and transferred to a polyvinylidene fluoride membrane (Immobilon-P, Millipore). The membrane was washed in Tris-buffered saline containing 0.05% Tween 20 (TBS-T) and blocked for 1 h at room temperature in TBS-T with 3% skim milk. The membrane was incubated overnight at 4°C either with an antibody (dilution 1:1000) for anti-Erk1/2 (#4696, Cell Signaling), anti-phosphorylated Erk1/2 (#4370, Cell Signaling), anti-JNK1/2/3 (#9926, Cell Signaling), or anti-phosphorylated JNK1/2/3 (#4668, Cell Signaling). The membrane was washed three times for 10 min with TBS-T and incubated with a secondary mouse anti-mouse IgG antibody conjugated to horseradish peroxidase (HRP, GE Healthcare, 1:6000) for 1 h at room temperature. The blots were detected with the Amersham ECL Western blotting detection reagent (GE Healthcare) and quantified with the Image J program (http://imagej.nih.gov/ij/index.html).

### Mouse model of sciatic nerve autograft

Adult male C57BL/6J mice (8 weeks old; 19.5 to 22.0 g) were purchased from Charles River. Mice were anesthetized with isoflurane and the left sciatic nerve was exposed through gluteal muscle incision under sterile conditions. The sciatic nerve was transected at two sites: (i) 3 mm distal to the first branch entering into biceps femoris (the proximal transected site) and (ii) 3 mm further distal to the proximal site (the distal transected site). The excised 3-mm nerve segment was moved aside to confirm complete dissection at two sites, then placed back to the original position. Perineuriums were sutured with 10–0 black nylon thread at the proximal and distal sites under microscopy. The remaining thread was later used as a marker to locate the transected sites when the mice were sacrificed. To reduce the toxicity of DMSO, we first made 60 mg/ml zonisamide in 100% DMSO, and then diluted it in olive oil to make 3 mg/ml zonisamide in 5% DMSO. Control solution (5% DMSO) or 30 mg/kg/day zonisamide (3 mg/ml x ~0.2 ml) was intragastrically administered using a disposable sonde needle (flexible type, Fuchigami) once a day starting from a day after surgery up to 1 or 8 weeks later when the mice were sacrificed. We analyzed myelination of sciatic nerves in six mice in each group. A 6-mm nerve segment was excised by cutting at the proximal transected site and at 3 mm distal to the distal transected site 1 week after the sciatic nerve autograft. Then the fibers were fixed in 2% paraformaldehyde/2.5% glutaraldehyde in 0.2 M sodium cacodylate buffer at pH 7.3 overnight at 4°C, postfixed in 2% osmium tetroxide for 3 h, and dehydrated in ascending concentrations of ethanol. After Epon embedding, semithin cross sections (1 μm) were obtained 0.7 mm distal to the distal transected site, stained with alkaline Toluidine blue (Sigma), and examined under a microscope (FSX100, Olympus). The number and area of myelinated axons in the sciatic nerve were manually counted using Image J software in a blinded manner.

Motor functions were assessed every week in three mice in each group by the walking track analysis, in which footprints of both hindlimbs were recorded when mice were allowed to walk freely on a runway [26]. Sciatic function index (SFI) was calculated by measuring the width and length of footprints of both legs in a blinded manner. If there is no difference between the cut and uncut hindlimbs, the SFI is 0, whereas the SFI is -100 when the cut hindlimb is completely paralyzed [27]. Before taking footprint records, the mice were trained to walk through the runway several times. Footprints were recorded on six to eight sheets each time. Eight weeks after the sciatic nerve autograft, the whole gastrocnemius muscle and a 3 mm sciatic nerve segment from the distal transected site to 3 mm further distal to it were isolated for extraction of total RNA. Similar specimens were isolated from the uncut hindlimb. Tibialis anterior muscles were also isolated and fixed with 100% cooled methanol for hematoxylin and eosin staining.

### Statistical analysis

Unpaired Student’s *t*-test, one-way or two-way ANOVA, and post-hoc Tukey honestly significant difference (HSD) test were performed by SPSS ver. 21 (IBM). *P* values of 0.05 or less were considered statistically significant.

## Results

### Zonisamide enhances neurite elongation of NSC34 motor neuron-like cells

To search for off-label effects of pre-approved drugs on neurite elongation of GFP-expressing NSC34 cells, we added 10 μM of 1,186 pre-approved drugs to NSC34 cells that were induced to differentiate into neurons. After 48 h, the average neurite lengths were automatically measured using the Cellomics Array Scan System. Each drug was tested four to sixteen times. A limited number of drugs after the first round of screening were added to primary motor neurons isolated from the spinal cords of C57/BL6 mice at E13.5. The cells were fixed after 48 h of culture, and the axons were fluorescently immunostained for Tau, followed by automatic measurement of the neurite lengths using the ArrayScan VTI HCS Reader. We found that zonisamide consistently elongated neurites of primary motor neurons (S1 Fig).

**Fig 1:**
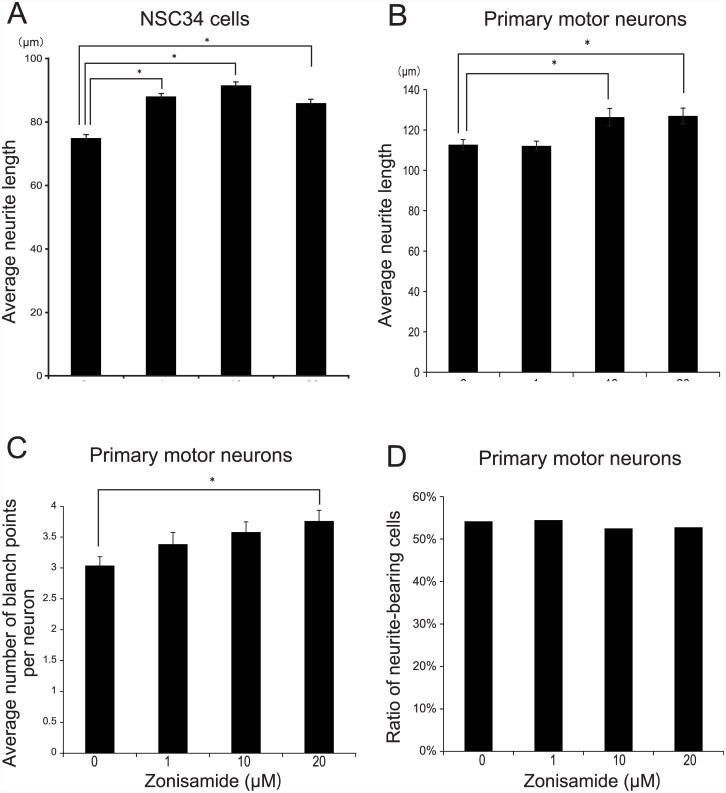
Zonisamide increases neurite length and the number of branch points in NSC34 cells and mouse primary spinal motor neurons. **(A, B)** Mouse NSC34 motor neuron-like cells **(A)** and primary motor neurons isolated from the spinal cord of mouse embryos at E13.5 **(B)** were differentiated for 48 h in the presence of the indicated concentrations of zonisamide. The neurite lengths of more than 200 cells were automatically measured with the ArrayScan. *p < 0.01 by one-way ANOVA followed by Tukey HSD. **(C)** The number of branch points of more than 200 differentiation-induced primary motor neurons was automatically measured with the ArrayScan. *p < 0.01 by one-way ANOVA followed by Tukey HSD. **(D)** The number of differentiation-induced primary motor neurons with neurite lengths of more than 20 μm was divided by the total number of cells under the microscope to calculate the ratio of neurite-bearing cells. No statistical significance was observed by one-way ANOVA. Mean and SE are indicated for all the panels.

We next looked into the dose response effects of zonisamide on neurite elongation of spinal motor neurons. We found that zonisamide enhanced axonal elongation in a concentration dependent manner in NSC34 cells (Fig 1A) and primary motor neurons (Fig 1B). Additionally, the number of branch points in the axons of primary motor neurons was also increased in a dose-dependent manner (Fig 1C). However, the outgrowth ratios of primary motor neurons, which were the ratios of neurite-bearing neurons, were not enhanced by zonisamide treatment (Fig 1D).

### Zonisamide enhances neurite regeneration of primary motor neurons

**Fig 2:**
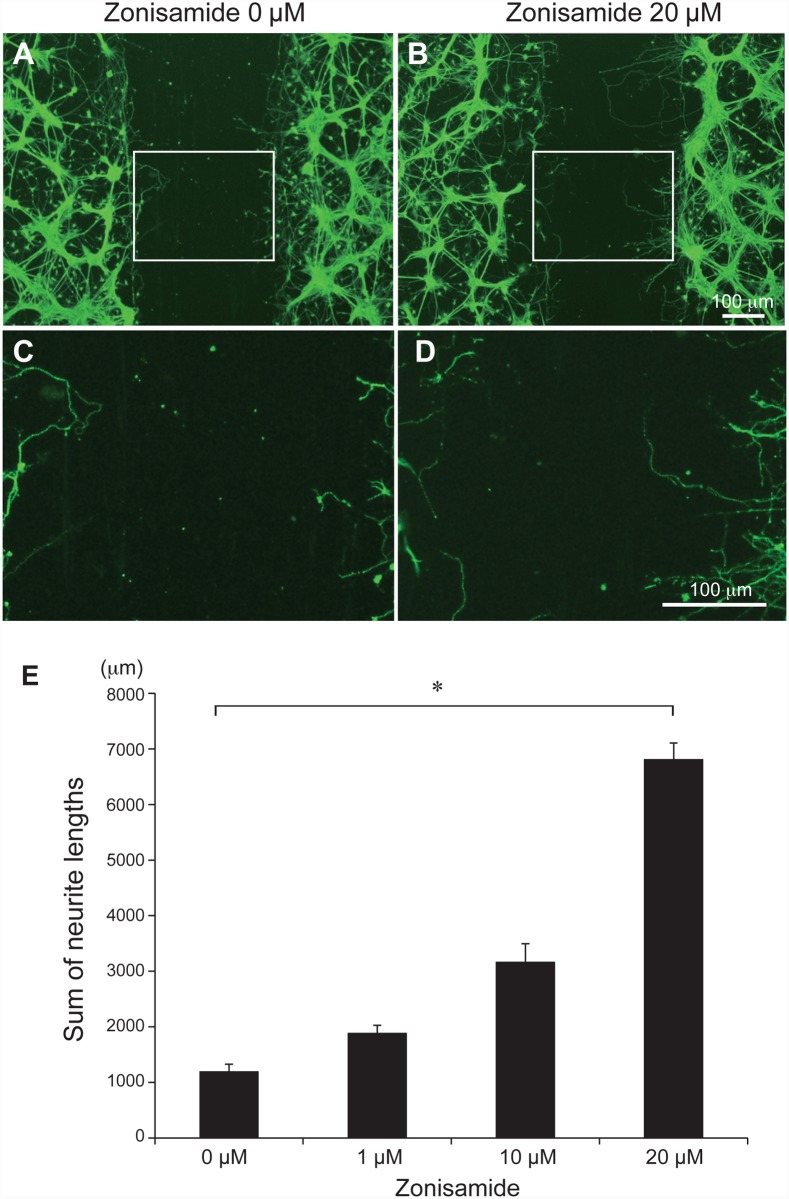
Zonisamide induces regeneration of neurites in primary motor neurons. **(A-D)** Differentiated primary motor neurons were scratched to evaluate their ability to regenerate neurites. Newly elongated neurite lengths into the scratched area after 48 h were automatically measured by the MetaMorph. Boxed areas in **(A)** and **(B)** are magnified in **(C)** and **(D)**. **(E)** The sum of regenerated neurite lengths in the square area (250 μm × 1000 μm) within the scratched area are calculated in four visual fields and the mean and SE are indicated. *p < 0.05 by one-way ANOVA followed by Tukey HSD.

To determine whether zonisamide enhances regeneration of axons, a scratch assay was conducted using mouse primary motor neurons. Primary motor neurons were differentiated for 48 h to extend neurites. A network of neurites on the culture plate was linearly scratched off with a 200-μl sterile pipette tip. Variable concentrations of zonisamide (0, 1, 10, or 20 μM) were added to the medium and the lengths of the neurites that elongated into the cutouts were automatically quantified with MetaMorph image analysis software. The lengths of the regenerated neurites elongated into the cutouts were increased in a dose-dependent manner (Fig 2). Zonisamide was thus able to enhance neurite regeneration in primary motor neurons.

### Zonisamide is unlikely to initiate neurite outgrowth, but is able to enhance neurite elongation in primary motor neurons

**Fig 3:**
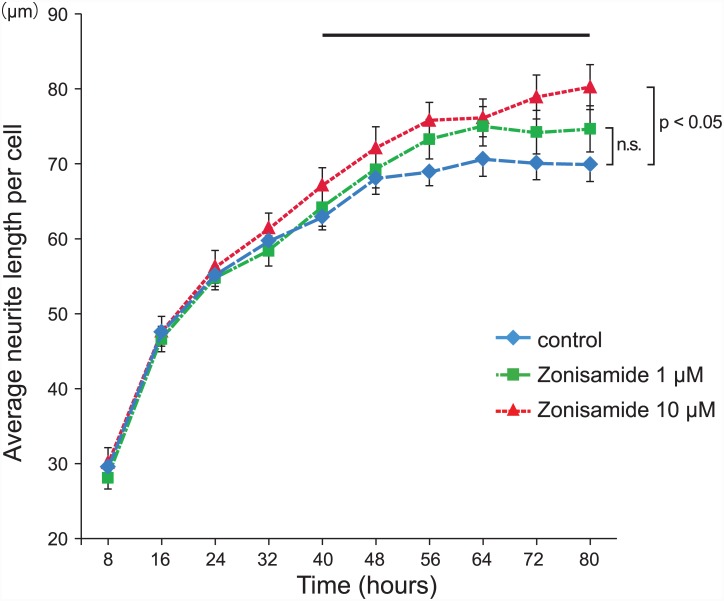
Temporal profile of zonisamide-induced neurite elongation of primary motor neurons. Average neurite lengths of differentiation-induced primary motor neurons were automatically measured every 8 h by the IncuCyte. Mean and SE of the average neurite length per cell in three culture dishes are indicated. The two-way ANOVA analysis was applied from 40 to 80 h (the bar on the top) after adding the differentiation medium. The statistical difference is indicated on the right side of the lines. n.s., not significant.

After zonisamide was administered to mouse primary motor neurons, the temporal profile of the axonal elongation was automatically traced with the IncuCyte ZOOM Live Cell Imaging System (Fig 3). Zonisamide had no overt effect up to 40 h after its administration. However, zonisamide-treated cells exhibited the neurite elongation effect after 40 h. We observed that 10 μM zonisamide had a greater effect than 1 μM throughout the observation period. These results suggest that zonisamide has an enhancing effect on neurite elongation rather than on the initiation of neurite outgrowth.

### Zonisamide is protective against oxidative stress in primary motor neurons

**Fig 4:**
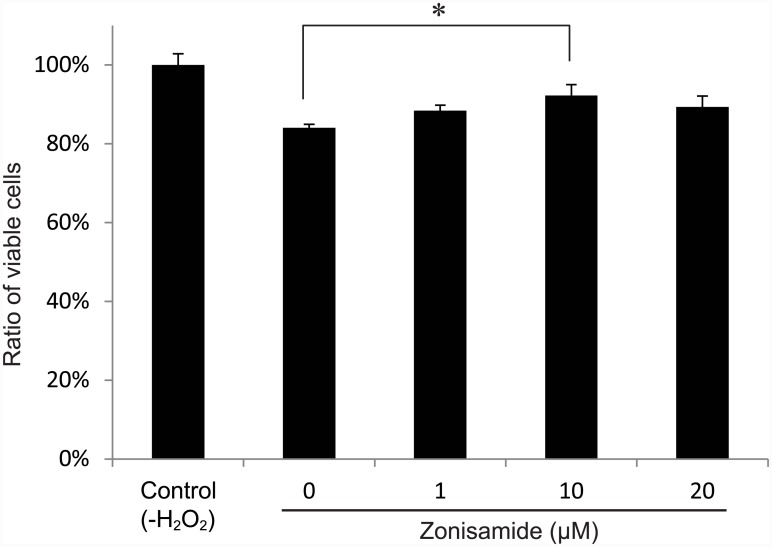
Zonisamide rescues cell death due to oxidative stress. Primary motor neurons in DMEM/F12 with 0.5% FBS were treated with variable concentrations of zonisamide. After 1 h, cells were exposed to 100 μM hydrogen peroxide (H_2_O_2_) for 24 h. The number of viable cells was estimated by the MTS assay and was normalized to that without H_2_O_2_ (control). Mean and SE are indicated (*n* = 6). *p < 0.05 by one-way ANOVA followed by Tukey HSD.

Primary motor neurons were treated with zonisamide for 1 h and then exposed to 100 μM H_2_O_2_ for 24 h to induce oxidative stress-mediated cell death. Subsequently, the number of viable primary motor neurons was quantified with an MTS assay. Zonisamide increased the number of viable cells in a dose-dependent manner (Fig 4). Zonisamide thus provided a neuroprotective effect against oxidative stress in primary motor neurons.

### Zonisamide upregulates expression of neurite elongation-related genes

**Fig 5:**
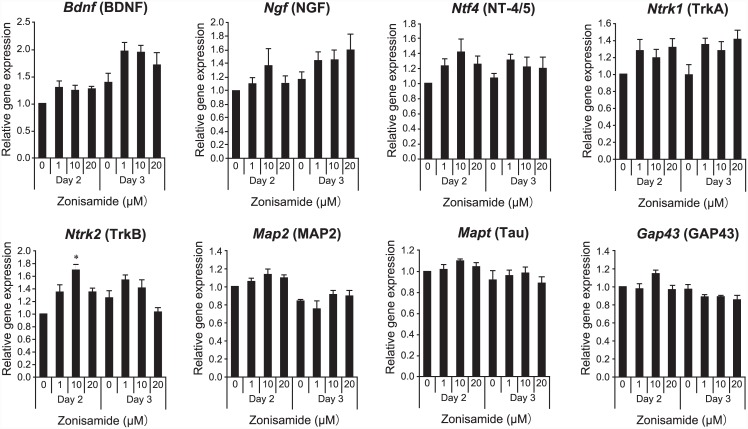
Effects of zonisamide on mRNA levels of nerve growth factors and their receptors, as well as neurite markers in primary motor neurons. Gene expression in primary motor neurons was quantified by quantitative RT-PCR on days 2 and 3 after adding the differentiation medium. The indicated concentration of zonisamide was added 8 h after adding the differentiation medium. Gene expression was normalized to *Gapdh* and to cells without zonisamide. Mean and SE are indicated (*n* = 4). *p < 0.05 by one-way ANOVA followed by Tukey HSD.

We added 0, 1, 10, or 20 μM zonisamide to primary motor neurons and evaluated mRNA expression levels by quantitative RT-PCR. Zonisamide increased the mRNA levels of the neurotrophins *Bdnf*, *Ngf*, and *Ntf4* on days 2 and 3, although dose-response effects were not always observed (Fig 5). The mRNA levels of the receptors *Ntrk1* (a receptor for NGF) and *Ntrk2* (a receptor for BDNF and NT4-4/5) were also upregulated by zonisamide, again with equivocal dose-response effects. mRNA levels of the structural proteins *Map2* (enriched in dendrites), *Mapt* (enriched in axons), and *Gap43* (enriched in the growth cone), however, were not upregulated by zonisamide up to day 3. These results suggest that zonisamide induces expression of neurotrophins and their receptors.

We also looked into phosphorylation of Erk1/2 and JNK1/2/3, and found that zonisamide suppressed phosphorylation of Erk1/2 in NSC34 cells and primary motor neurons (S2 Fig). In contrast, zonisamide minimally enhanced phosphorylation of JNK1/2/3 in NSC34 cells, but had no effect in primary motor neurons (S2 Fig).

### Zonisamide facilitates nerve regeneration in a mouse model of sciatic nerve autograft

**Fig 6:**
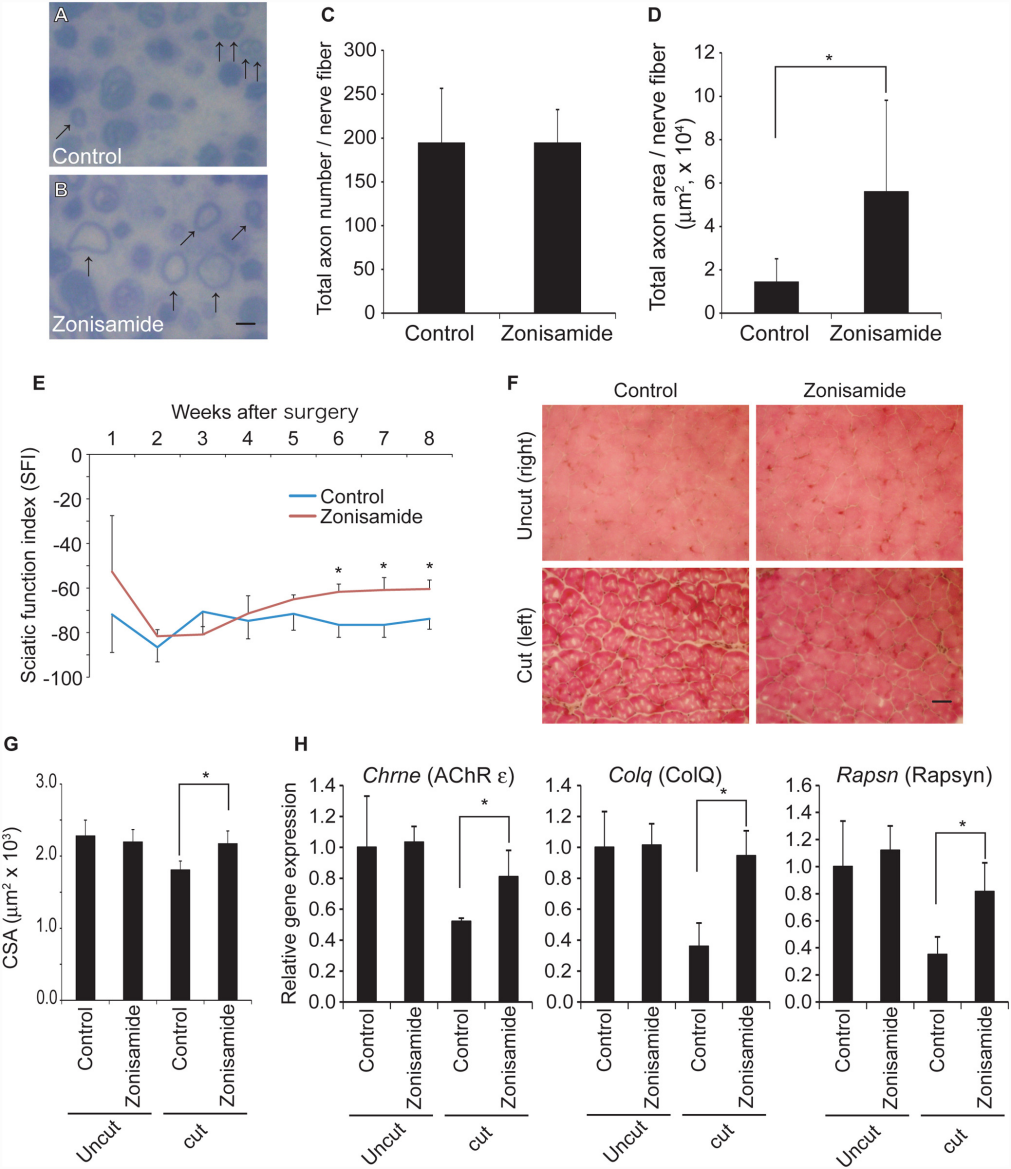
Effects of zonisamide on a mouse model of sciatic nerve autograft. Model mice were made by cutting the left sciatic nerve at two sites 3 mm apart. Mice (*n* = 9 in each group) were intragastrically given either control solution or 30 mg/kg/day zonisamide every day from 1 day after surgery up to 1 week **(A-D)** or 8 weeks **(E-H)** when mice were sacrificed. **(A, B)** Six mice in each group were scarified 1 week after surgery. Representative images of Toluidine blue-stained cross sections of the sciatic nerve 0.7 mm distal to the distal transected site are shown. Myelinated axons are indicated by small arrows. Bar = 20 μm. **(C, D)** Blinded morphometric analysis of the number **(C)** and area **(D)** of myelinated axons in a single sciatic nerve preparation 0.7 mm distal to the distal transected site. Mean and SD are indicated (*n* = 6 mice in each group). *p < 0.05 by Student’s *t*-test. **(E)** Temporal profile of sciatic function indices (SFIs) of footprints of mice treated with control solution or 30 mg/kg/day zonisamide every day from 1 day after surgery. Mean and SD of three mice in each group are indicated. Note that the SFIs are improved in zonisamide-treated mice from 6 weeks after sciatic nerve autograft. *p < 0.05 by Student’s *t*-test. **(F)** Representative images of hematoxylin and eosin staining of cross sections of tibialis anterior 8 weeks after surgery. Note fewer target fibers and larger fiber sizes in zonisamide-treated tibialis anterior. Bar = 30 μm. **(G)** Blinded morphometric analysis of cross-sectional areas (CSA) of muscle fibers of tibialis anterior 8 weeks after surgery. Mean and SD are indicated (*n* > 98 in each group). *p < 0.05 by Student’s *t*-test. **(H)** Quantitative RT-PCR of *Chrne*, *Colq*, and *Rapsn* expression in gastrocnemius muscles with uncut and cut sciatic nerves 8 weeks after surgery. Gene expression normalized to *Gapdh* on the uncut right side of mice taking control solution was set at 1.0, and relative gene expression is indicated by mean and SD (*n* = 3 in each group). *p < 0.05 by Student’s *t*-test.

To examine the effects of zonisamide on nerve regeneration *in vivo*, we made a mouse model of sciatic nerve autograft by transecting and suturing the left sciatic nerve at two sites that were 3 mm apart. Zonisamide (30 mg/kg/day) was intragastrically administered to the model mice every day from a day after surgery. One week after surgery, analysis of a cross section of the sciatic nerve 0.7 mm distal to the distal transected site demonstrated that the number of axons remained unchanged but the area of axons was increased 3.9-fold (Fig 6A–6D). We also looked into the long-term effects of zonisamide on motor functions and the targeted muscles (tibialis anterior and gastrocnemius). The sciatic function index (SFI), a marker for motor function of hindlimbs after sciatic nerve autograft [27], was significantly improved from 6 to 8 weeks after surgery in zonisamide-treated mice (Fig 6E). Eight weeks after surgery, histological and morphometric analyses of cross sections of tibialis anterior muscle showed that zonisamide accelerated recovery from denervation atrophy (Fig 6F and 6G). In addition, gene expression of *Chrne* [28], *Colq* [29], and *Rapsn* [30], which are specifically expressed at the neuromuscular junction, were increased in zonisamide-treated mice (Fig 6H). We also examined gene expression in a sciatic nerve segment distal to the distal transected site (S3 Fig). Eight weeks after surgery, mRNA levels of the neurotrophins *Ngf* and *Ntf4*, but not *Bdnf*, were increased in zonisamide-treated mice (S3 Fig). mRNA levels of their receptors *Ntrk1* and *Ntrk2* were also increased by zonisamide, although gene expression of *Ntrk1* was low and variable in both uncut and cut hindlimbs (S3 Fig). We analyzed the same genes in primary motor neurons, and observed similar upregulation of neurotrophins and their receptors by zonisamide (Fig 5). In contrast to primary motor neurons, mRNA levels of the structural proteins *Map2*, *Mapt*, and *Gap43* were increased in model mice (S3 Fig). Taken together, our mouse model showed that zonisamide enhanced axonal regeneration and functional recovery of the sciatic nerve.

## Discussion

Here, we identified that the widely prescribed anti-epileptic and anti-Parkinsonian drug, zonisamide, enhanced neurite elongation in NSC34 cells and primary motor neurons. In addition, zonisamide increased the number of axonal branch points, enhanced neurite regeneration, and rescued H_2_O_2_-induced cell death of primary motor neurons. We also confirmed that zonisamide improved nerve regeneration and functional recovery in a mouse model of sciatic nerve autograft. Zonisamide is an anti-epileptic agent widely used for adjunctive treatment of partial seizures in adults and is an effective anti-parkinsonian agent especially for resting tremor [31]. The antiepileptic effects of zonisamide are accounted for by inhibition of sodium channels [32], inhibition of T-type calcium channels [33, 34], indirect inhibition of glutamate receptors [35], suppression of GABA/benzodiazepine receptors [36, 37], and enhancement of release of the inhibitory neurotransmitter GABA [36, 37]. In addition, zonisamide has other pharmacological effects, such as protective effects for dopaminergic neurons [38], neuroprotection against cerebral ischemia [39], an anti-oxidant effect in epilepsy [40], and a radical scavenging action [41, 42]. Furthermore, zonisamide attenuated degeneration of motor neurons and loss of astrocytes in a model mouse of amyotrophic lateral sclerosis [43], although the underlying mechanisms remain unsolved. The effect of zonisamide on sparing motor axons, however, has not been examined to date.

We found that mRNA levels of neurotrophins (*Bdnf*, *Ngf*, and *Ntf4*) and their receptors (*Ntrk1* and *Ntrk2*) were upregulated by zonisamide in primary motor neurons and in model mice. A recent report showed that TrkB agonists promoted axonal regeneration in a mouse model of peripheral nerve injury [44]. Enhanced expression of *Bdnf* and *Ntrk2* by zonisamide may partly account for the enhanced neurite elongation in primary motor neurons and enhanced axonal regeneration in model mice. Activation of Erk1/2 [45, 46] and JNK1/2/3 [47, 48] are observed in neurite outgrowth. The attenuation of Erk1/2 phosphorylation by zonisamide and lack of effect on JNK1/2/3 phosphorylation by zonisamide in primary motor neurons were thus unexpected. In addition to neurite outgrowth, activation of Erk is also observed in apoptosis of neuroblastoma cells [49]. Consistent with the role of Erk in apoptosis, inhibition of Erk activation by a MEK inhibitor protected against focal cerebral ischemia [50, 51] as well as against oxidative stress in a mouse neuronal cell line and rat primary cortical neurons [52]. Accordingly, the attenuated phosphorylation of Erk1/2 by zonisamide that we observed might have conferred the neuroprotective effect in H_2_O_2_-treated primary motor neurons.

Gene expression of structural proteins (*Map2*, *Mapt*, and *Gap43*) were not upregulated by zonisamide in primary motor neurons, but were increased in model mice. Similarly, zonisamide upregulated genes specifically expressed at the neuromuscular junction (*Chrne*, *Colq*, and *Rapsn*). We observed the effects of zonisamide for up to 3 days in primary motor neurons. In contrast, we observed the effects of zonisamide for 8 weeks in model mice. Three days were likely to be sufficient to observe the effects of zonisamide on neurotrophins and their receptors, but this period was too short to observe the effects on structural proteins.

The maximum clinically used dose of zonisamide for patients with epilepsy is 600 mg/day. Assuming that the human body weight is 60 kg, 600 mg/day is equivalent to 10 mg/kg/day. We used three times more zonisamide (30 mg/kg/day) for the model mice. The human threshold of maximum dosage, the TDI (tolerable daily intake), is extrapolated from the rodent threshold, the NOAEL (no observed adverse effect level), by dividing the NOAEL by the UF (uncertainty factor) [53]. As the default UF value is 10 [54, 55], the three times greater dosage of zonisamide that we used in the mouse model will be readily extrapolated to human studies. As zonisamide has long been used without major adverse effects, we hope that zonisamide is a clinically applicable therapeutic drug for peripheral nerve injuries and neuropathies due to other etiologies, although additional animal and human studies are required.

## Supporting Information

S1 Fig Effects of zonisamide on neurite elongation of NSC34 cells and primary motor neurons. Average neurite lengths in 16 wells are normalized to those of controls and are individually plotted. Mean and SD are indicated. Neurite lengths were automatically estimated by the ArrayScan VTI HCS Reader.
(EPS)

S2 Fig Western blotting of phosphorylated and total Erk1/2 in NSC34 cells (A) and primary motor neurons (B), as well as phosphorylated and total JNK1/2/3 in NSC34 cells (C) and primary motor neurons (D). Representative blots of three or more experiments are shown. Ratios of phosphorylated to total Erk1/2 and JNK1/2/3 are normalized to those of the controls and are indicated below the blots.
(EPS)

S3 Fig Effects of zonisamide on mRNA levels of nerve growth factors and their receptors, as well as neurite markers in sciatic nerve in a mouse model of sciatic nerve autograft. Quantitative RT-PCR of the indicated genes in a sciatic nerve segment from the distal transected site to 3 mm further distal to it 8 weeks after sciatic nerve autograft. Gene expression normalized to *Gapdh* on the uncut right side of mice taking control solution was set at 1.0, and relative gene expression is indicated by mean and SD (*n* = 3 in each group). *p < 0.05 by Student’s *t*-test. Note that all gene expression tended to be increased with zonisamide treatment on the autograft side, except for *Bdnf* and *Ntrk1*, for which gene expression was low and variable.
(EPS)

S1 Table Primer sequences for quantitative RT-PCR. (DOCX)

## Abbreviations

FDA: Food and Drug Administration
NGF: nerve growth factor
BDNF: brain-derived neurotrophic factor
HCS: high content screening
GABA: gamma-aminobutyric acid
