# Correction: Zonisamide Enhances Neurite Elongation of Primary Motor Neurons and Facilitates Peripheral Nerve Regeneration *In Vitro* and in a Mouse Model

**DOI:** 10.1371/journal.pone.0148470

**Published:** 2016-01-29

**Authors:** Hideki Yagi, Bisei Ohkawara, Hiroaki Nakashima, Kenyu Ito, Mikito Tsushima, Hisao Ishii, Kimitoshi Noto, Kyotaro Ohta, Akio Masuda, Shiro Imagama, Naoki Ishiguro, Kinji Ohno

Fig 4 is incorrect. The authors have provided a corrected version here.

**Fig 4:**
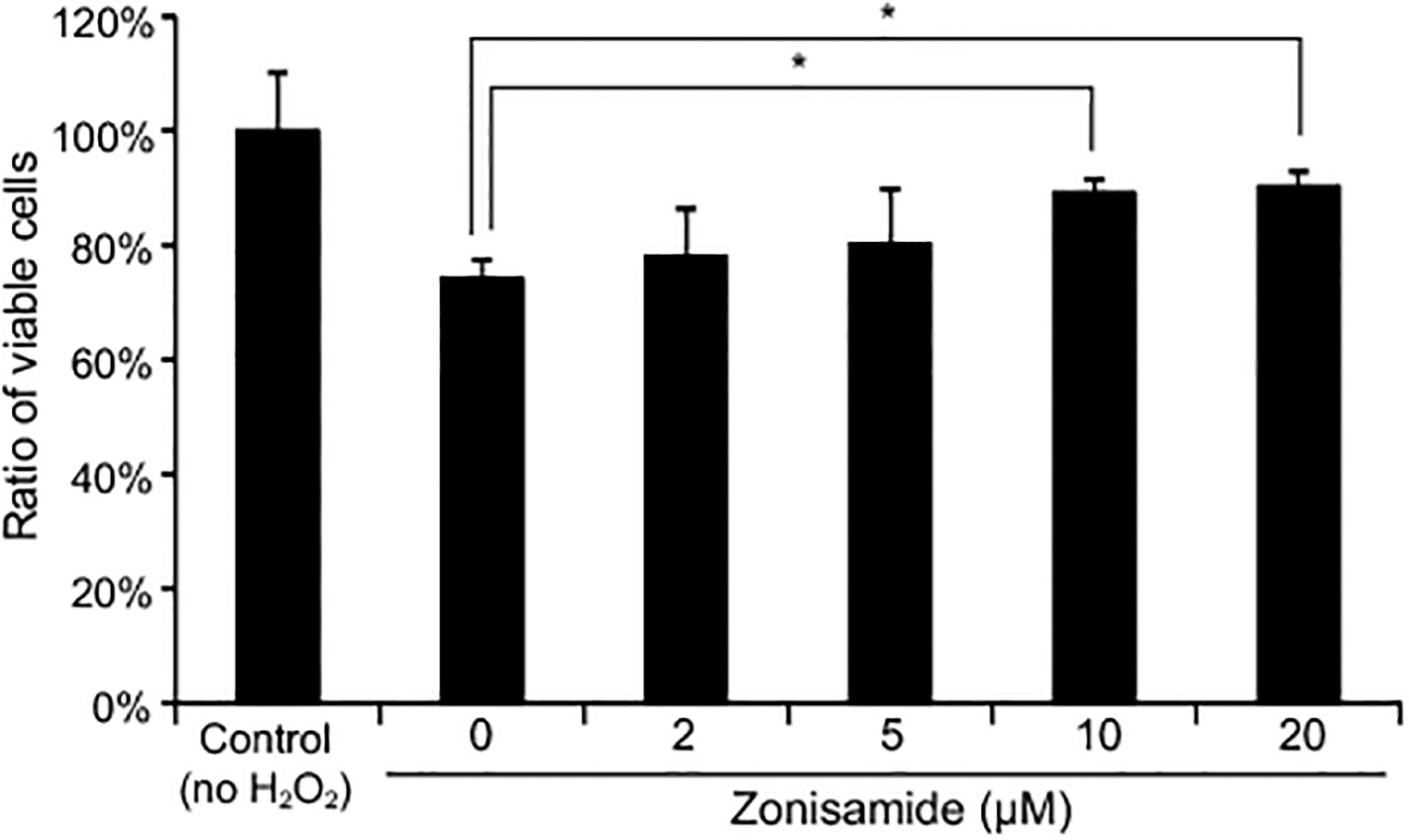
Zonisamide rescues cell death due to oxidative stress. Primary motor neurons in DMEM/F12 with 0.5% FBS were treated with variable concentrations of zonisamide. After 1 h, cells were exposed to 100 μM hydrogen peroxide (H_2_O_2_) for 24 h. The number of viable cells was estimated by the MTS assay and was normalized to that without H_2_O_2_ (control). Mean and SE are indicated (n = 6). *p < 0.05 by one-way ANOVA followed by Tukey HSD.
