# Supplementary material for: Zonisamide Enhances Neurite Elongation of Primary Motor Neurons and Facilitates Peripheral Nerve Regeneration In Vitro and in a Mouse Model
Source: PLoS One. 2015 Nov 16;10(11):e0142786. doi: 10.1371/journal.pone.0142786 (PMC4646494; doi:10.1371/journal.pone.0142786)
Supplement: S1 Table — (DOCX) [file pone.0142786.s004.docx]

**Table S1. Primer sequences for quantitative RT-PCR**

| **Target gene** | Forward | | Reverse |
| --- | --- | --- | --- |
| ***Gapdh*** | 5'-ACCCCUUCAUUGACCUCAAC-3’ | 5'-UCCCGUUGAUGACAAGCUUC-3’ | |
| ***Bdnf* (BDNF)** | 5'-TTGTTTTGTGCCGTTTACCA-3’ | 5'-GGTAAGAGAGCCAGCCACTG-3’ | |
| ***Ngf* (NGF)** | 5'-CCTCCAATCCTGTTGAGAGTG-3’ | 5'-TGTGAGTCGTGGTGCAGTATG-3’ | |
| ***Ntf4* (NT-4/5)** | 5'-CCCTGCGTCAGTACTTCTTCGAGAC-3’ | 5'-CTGGACGTCAGGCACGGCCTGTTC-3’ | |
| ***Ntrk1* (TrkA)** | 5'-ATCTAGCCAGCCTGCACTTTGT-3’ | 5'-TCTGCTCATGCCAAAGTCTCC-3’ | |
| ***Ntrk2* (TrkB)** | 5'-TGACCCACTCCCCACCTTG-3’ | 5'-TCGACTCCAGGCCGGCCCATG-3’ | |
| ***Map2* (MAP2)** | 5'-CTGGACATCAGCCTCACTCA-3’ | 5'-AATAGGTGCCCTGTGACCTG-3’ | |
| ***Mapt* (Tau)** | 5'-TGAGGGACTAGGGCAGCTAA-3’ | 5'-CAGTCCACCCATCCATCTCT-3’ | |
| ***Gap43* (GAP43)** | 5'-GGCTCTGCTACTACCGATGC-3’ | 5'-GGCTTGTTTAGGCTCCTCCT-3’ | |
| ***Chrne* (AChR ε)**  ***Colq* (ColQ)**  ***Rapsn* (rapsyn)** | 5'-GCCCTGCTTCTCCTGACACTCTTTG-3’  5'-AAGCAGGGTCAGAAGGGAGACAGTG-3’  5'-TGGGGCAGGACCAGACAAAGCAAC-3’ | 5'-TCGTCCTTGCTGTAGTTGAGCCG-3’  5'-TTCACATTCACAGGGGGTCCGC-3’  5'-TCCAGTCCCCGAGCAGTATCAATC-3’ | |
